# Development of functional spatial mapping of the tumour immune microenvironment (FuncO:TiME) in advanced melanomas

**DOI:** 10.1038/s44276-026-00250-w

**Published:** 2026-09-30

**Authors:** Emma Wagner, Samuel Legg, Christopher J. Applebee, Amanda R. Kirane, Julian Padget, Banafshé Larijani

**Affiliations:** 1 Division of General Surgery, Department of Surgery, Section of Surgical Oncology, Stanford University School of Medicine, Stanford, CA USA; 2 Department of Computer Science, University of Bath, Bath, UK; 3 Cell Biophysics Laboratory, Department of Life Sciences, Consortium for Precision Health, University of Bath, Bath, UK; 4 Department of Computer Science, Consortium for Precision Health, University of Bath, Bath, UK

## Abstract

**Introduction:**

Current spatial proteomic approaches quantify immune checkpoint expression but do not directly measure functional receptor-ligand (PD-1/PD-L1) interactions within the tumour-immune microenvironment (TiME). Therapeutic antibodies disrupt receptor-ligand interactions and do not target protein abundance; therefore, methods capable of resolving functional checkpoint interactions may provide biologically distinct insight beyond expression-based assays.

**Methods:**

We developed FuncO:TiME by integrating functional oncology mapping (FuncOmap), histopathology and multiplex spatial immune profiling to map PD-1/PD-L1 interaction states within clinically annotated melanoma tissues collected before and after neoadjuvant immune checkpoint blockade (ICB).

**Results:**

FuncOmap quantified millions of per-pixel PD-1/PD-L1 interactions and revealed marked spatial heterogeneity in checkpoint interactions that were not reflected by PD-1 or PD-L1 expression alone. Post-treatment tissues exhibited increased PD-1/PD-L1 interaction states despite no corresponding increase in expression, highlighting the divergence between functional checkpoint interactions and expression-based measurements. Integrating with spatial immune profiling further demonstrated the feasibility of contextualising regional differences in interaction states within distinct immune populations and patterns of PD-1/PD-L1 expression in melanoma TiMEs.

**Conclusion:**

We establish the feasibility of FuncO:TiME spatially resolved mapping of functional checkpoint interactions in human melanoma tissues. Our findings demonstrate that receptor-ligand interactions diverge from checkpoint protein expression patterns and support further development of integrated approaches that contextualise functional checkpoint interactions within cellular features of the TiME. Such approaches may enable deeper investigation of checkpoint biology and inform the future development of functionally informed biomarkers for cancer immunotherapy.

## Introduction

Immune checkpoint blockade (ICB) has transformed the treatment landscape for advanced melanoma, yet clinical response remains heterogeneous, and robust biomarkers to guide therapeutic decision-making remain limited. Current tissue-based approaches, including digital spatial proteomics and transcriptomics, primarily quantify protein and RNA expression within the tumour-immune microenvironment (TiME) [1–3]. While these methods have provided important insight into immune composition and checkpoint protein abundance, expression levels alone do not directly measure functional receptor-ligand interactions (e.g. downstream signalling impacted by molecular and conformational changes and post-translational modifications). Therapeutic antibodies target protein interactions rather than protein abundance; therefore, inferring functional checkpoint interactions from expression-based metrics may obscure critical aspects of immune regulation, particularly in the context of ICB therapy [4, 5].

Time-resolved Förster resonance energy transfer (FRET) acquired using fluorescence lifetime imaging microscopy (FLIM) quantifies intra- and intercellular receptor-ligand interactions within 1–10 nm and has previously demonstrated that functional immune checkpoint interaction states can diverge from protein expression levels in multiple solid tumour types [6–11]. The spatial resolution of intra- and intercellular time-resolved FRET (aiFRET) has been further advanced through the development of FRET-based functional oncology mapping (FuncOmap), which directly quantifies and spatially localises protein interaction states in fixed human tissues at per-pixel resolution [6, 10, 12–15].

These aiFRET and FuncOmap studies underscore the conceptual distinction between checkpoint expression and checkpoint interaction and how the latter may associate with clinical outcomes more closely than abundance metrics alone [7, 10–16]. Supplementary Table 1 provides a comparative overview of the advantages and limitations of aiFRET and FuncOmap relative to other antibody-based assays commonly used to quantify immune checkpoint interactions, including proximity ligation assay (PLA) and immunohistochemistry (IHC). Although FuncOmap precisely quantifies receptor-ligand interaction states, it does not contextualise these interactions within defined TiME cell populations. Conversely, digital spatial profiling (DSP) platforms provide multiplexed, spatially resolved protein expression data that enable characterisation of tumour and immune cell types expressing PD-1 and PD-L1, but lack the resolution required to determine which TiME cell populations contribute to functional interactions [1–3].

To address these caveats, we developed FuncO:TiME, an imaging and computation framework that maps multiple-frequency-domain FLIM (mfFLIM)-based FuncOmaps and DSP to Hematoxylin & Eosin (H&E)-stained tissue sections to enable spatial mapping of PD-1/PD-L1 interaction states within clinically annotated melanoma tissues. This integration correlates per-pixel quantification of functional interactions with spatial immune cell characterisation, permitting contextualisation of checkpoint interaction intensity within distinct immune cell populations of the TiME.

In this methodological and feasibility study, we apply FuncO:TiME to melanoma specimens collected before and after neoadjuvant ICB. We demonstrate that PD-1/PD-L1 interaction states exhibit marked spatial heterogeneity that is not reflected by PD-1 or PD-L1 expression alone and illustrate post-treatment divergence in interaction patterns across clinically annotated response groups. Advanced spatial profiling of pre-treatment tissue from a complete responder demonstrated that intratumoural variation in PD-1/PD-L1 interactive states was associated with distinct immune cell-specific PD-1/PD-L1 expression patterns. These findings establish FuncO:TiME as a framework for spatially resolved functional checkpoint mapping in human tumours and provide a foundation for future studies investigating checkpoint interaction dynamics in larger patient cohorts.

## Methods and materials

### Patient samples

Formalin-fixed paraffin-embedded (FFPE) slides collected from patients with advanced melanoma were used in this study. Samples were collected prior to standard therapies and at the time of interval treatment assessment. All samples and clinical data were collected with the approval of Stanford University’s Institutional Review Board (IRB). Since this study was designed as a methodological feasibility investigation, formal power calculations were not performed. Patient response outcomes were blinded and only revealed when analyses were finalised. The clinicopathologic features of patients used in this study are summarised in Supplementary Table 2.

### Antibodies and reagents

See Supplementary Methods and Materials for a full list of antibodies used in FuncOmap and COMET assays.

### FuncOmap two-site assay for PD-1/PD-L1 functional interactions

This follows already published methodology [10]. More details about the two-site assay and the semi-automated non-biased acquisition of the lifetime maps (delivering technical reproducibility) are provided in Supplementary Methods and Materials.

### FuncO:TiME computational framework workflow

The Framework workflow is depicted in Supplementary Fig. 1. FLIM-derived FRET efficiency (Ef) maps were generated using the FuncOmap software pipeline [10], in which each pixel represents the calculated Ef value corresponding to PD-1/PD-L1 interaction. PD-1/PD-L1 interactive states were mapped across entire tissue sections via 77 non-overlapping 512 × 512-pixel Ef maps. Each Ef map is automatically thresholded by the FuncOmap computational algorithm to ensure unbiased selection of regions where PD-1/PD-L1 Ef values are above background levels [10].

Ef maps were computationally aligned to clinically annotated H&E sections to enable spatial comparison between functional checkpoint interactions and histologic features. Alignment was performed using a five-step computational procedure. First, sampling paths connecting FLIM acquisition regions were reconstructed from microscope stage position metadata. Second, Otsu thresholding was applied to the H&E image to generate a tissue mask. Third, global scaling, rotation and translation were applied to maximise overlap between FLIM sampling regions and the H&E tissue mask. Fourth, local optimisation was performed around each sampling region individually within a 200-pixel window (corresponding to approximately 10% of the H&E image dimensions). Finally, regions were identified where H&E tissue signal spatially coincided with acceptor fluorescence intensity (PD-L1) measured by FuncOmap. Consequently, the user is presented with a map of points overlaid on the H&E image. Clicking on a point opens a heatmap of the variations in Ef for that region. On this heatmap, using the system’s pseudo-colourmap, high Ef areas show red, while lower-intensity regions transition through light blue and yellow to red. In addition, for each 512 × 512-pixel coincidence FLIM image frame, 1000 datapoints were randomly sampled from 2.6 × 10^6^ total datapoints to generate mini violin plots representing the local distribution of Ef values. Sampling stability was evaluated by generating three independent random samples per region and confirming that their distributions were comparable. The resulting spatial map enables visualisation of region-specific Ef distributions overlaid on histologic architecture. The code supporting image registration and interaction mapping will be made available upon reasonable academic request through the University of Bath.

In this study, FuncOmap regions (Ef 1–10) were selected as an example to compare how PD-1/PD-L1 Ef values can vary across adjacent tumour-positive (Ef 1–5) and tumour-negative (Ef 6–10) tissue regions and generate hypotheses of how differences in tissue architecture may impact baseline PD-1/PD-L1 interactive states. Ef regions 1–10 are not meant to be used as representative Ef values for entire tissue. To do so would require users to consider additional information, including population densities of per-pixel Ef values across whole- and user-defined tissue regions, which is possible via FuncO:TiME computational workflow (Supplementary Fig. 1).

### Statistical analyses

Violin plots intrinsically demonstrate the distribution of numerical data and are particularly effective for comparing multiple groups side-by-side. Hence, violin plots were used to quantify the distribution of Ef values across tissue regions and samples.

#### Mini violin plots

1000 datapoints were randomly sampled from the total 2.6 × 10^6^ datapoints generated from each 512 × 512-pixel coincidence FLIM image frame as described above. This sample was used to generate a mini violin plot which provides a local approximation of the true distribution of Ef. The representativeness of the random samples was verified by generating three sample sets and checking that the variance between them was low.

#### Global violin plots

The global violin plots were generated from all 512 × 512-pixel coincident images, corresponding to approximately 30 × 10^6^ datapoints per global violin plot. The median and upper quartile (Q3) of the global Ef distribution was used as a descriptive metric to represent regions with the highest PD-1/PD-L1 interaction states across tissues. Ef distributions are non-Gaussian; quartile-based summaries were used to quantify differences between samples.

### Antigen retrieval and COMET seqIF imaging

Briefly, FFPE slides were de-waxed, and antigen retrieval was performed by placing the slides in Dewax and HIER Buffer H (Epredia, catalogue number: TA-999-DHBH) inside the PT Module (Epredia, catalogue number: A80400011) for 20 min at 95 °C. Processed slides were immediately placed into Multi-staining Buffer (Lunaphore Technologies SA, catalogue number: BU06) until use. Slides were transferred to the COMET instrument, where a microfluidic COMET Chip (Lunaphore Technologies SA, catalogue number: MK03) was sealed on top of the processed FFPE tissue section by the COMET instrument to form a closed reaction chamber. FFPE tissue sections were stained by reagents delivered via the microfluidic channels according to the pre-loaded seqIF protocols. Automated seqIF protocols were generated using the COMET Control software and consisted of iterative autofluorescence, staining, imaging and elution cycles [17]. Further details are provided in Supplementary Methods and Materials.

### COMET seqIF image processing, nuclear detection and cell segmentation

COMET seqIF OME-TIFF images were background-corrected using HORIZON™ Viewer software (Lunaphore Technologies SA) and subsequently imported into QuPath (v0.6.0) for image analysis. Tumour-positive (tumour) versus tumour-negative (non-tumour) tissue regions and FuncOmap regions (Ef 1–6) from clinically annotated H&E-stained tissue sections (see “FuncO:TiME computational framework workflow” section) were manually transferred onto COMET seqIF images in QuPath. As H&E staining, FuncOmap and COMET seqIF were performed on sequential tissue sections, corresponding tumour and FuncOmap region annotations were manually transferred onto COMET seqIF images in QuPath using tissue architecture and histopathological landmarks rather than direct image registration.

Nuclei in COMET seqIF images were detected using the StarDist pre-trained fluorescence model (dsb2018_heavy_augment), and whole-cell boundaries were estimated using QuPath’s cell expansion workflow. For each segmented cell, QuPath extracted centroid coordinates, morphological measurements and mean fluorescence intensity values for all markers included in the COMET seqIF panel. Cell detection results were exported as tab-delimited text files containing cell metadata, spatial coordinates, tumour and FuncOmap region annotations, and marker intensity measurements for downstream analyses in RStudio (v4.5.2).

### COMET seqIF and FuncO:TiME spatial analyses

Single-cell COMET seqIF data were imported into RStudio and marker fluorescence intensities were z-score normalised across all cells on a marker-by-marker basis. Immune lineages were assigned using a biologically informed scoring approach based on lineage-defining marker combinations. For each cell, lineage scores were calculated as the mean z-score normalised expression of relevant markers: melanoma (HMB45, SOX10), tumour-associated macrophages (TAM; CD68), T cells (CD3), CD4^+^ T cells (CD3, CD4), CD8^+^ T cells (CD3, CD8), regulatory T cells (CD3, CD4, FOXP3), B cells (CD20), natural killer (NK) cells (CD56), myeloid cells (CD11b), monocytes (CD14, CD11b), dendritic cells (CD11c, HLA-DR, MHCII), neutrophils (CD15, CD16), and myeloid-derived suppressor cells (MDSC; CD15).

Each cell was assigned to the lineage with the highest lineage score, provided the maximum lineage score exceeded zero, thereby resolving cells expressing markers associated with multiple immune lineages. Cells lacking a positive lineage score were classified as “Other”. For downstream visualisation, individual lineage annotations were collapsed into broad categories comprising Melanoma, TAM, Monocyte/Myeloid, Dendritic, Total T cells, NK, B cell, MDSC/Neutrophil and Other. Total T cells included CD4 T cells, CD8 T cells, regulatory T cells (Tregs) and unclassified T cells. PD-1-positive and PD-L1-positive cells were defined as cells with z-score normalised PD-1 or PD-L1 fluorescence intensities greater than zero, corresponding to expression above the dataset mean for the respective marker.

TAM phenotypes were characterised using a continuous TAM balance score calculated as the difference between composite M1 and M2 scores (M1 − M2). M1 scores were calculated as the mean z-score normalised expression of CD86, HLA-DR and MHCII, whereas M2 scores were calculated as the mean z-score normalised expression of CD163 and CD206. TAMs with positive balance scores were classified as M1-like TAMs, whereas TAMs with negative balance scores were classified as M2-like TAMs. Broad immune lineage composition, TAM and T-cell phenotypes, and the distribution of PD-1-positive and PD-L1-positive cells across FuncOmap regions were visualised using stacked bar plots generated with ggplot2. For each FuncOmap region, the proportion of PD-1-positive and PD-L1-positive cells within each annotated lineage was calculated and visualised as percentage-based heatmaps generated with ggplot2.

### Ethics approval and consent to participate

Stanford University, CA, USA - Institutional Review Board (IRB) and Certification of Human Subjects approval; March 19th, 2026; Awarded to Dr Amanda Robinson Kirane, MD, Surgery - General Surgery at Stanford University; eProtocol #: 65607; IRB 5; Registration 4593; The IRB and Certification of Human Subjects, and consent were obtained from all participants. All methods in the manuscript were performed in accordance with the relevant guidelines and regulations of the IRB.

## Results

This study was designed as a methodological feasibility analysis to develop and evaluate FuncO:TiME as a computational framework that integrates histological annotation (H&E), functional interaction mapping (FuncOmap), and DSP to contextualise PD-1/PD-L1 interactions within distinct tumour regions and immune populations of the TiME.

### FuncOmap spatially quantifies PD-1/PD-L1 molecular engagement rather than inferring PD-1/PD-L1 proximity from protein abundance

**Fig. 1:**
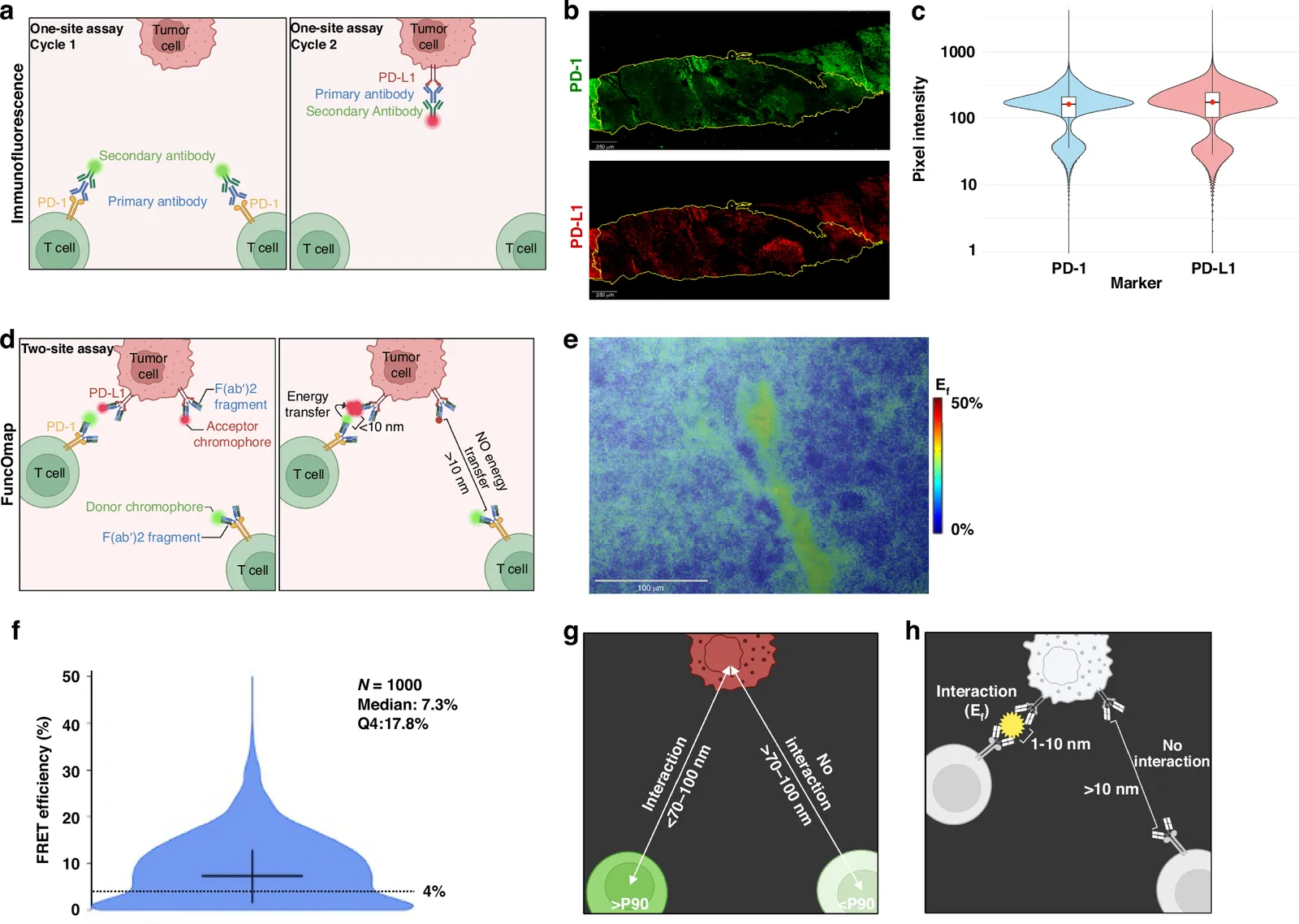
Spatial quantification of PD-1/PD-L1 interactions using one-site assays versus FuncOmap. **a** Schematic depiction of a conventional one-site immunofluorescence assay; PD-1 and PD-L1 are labelled using a primary antibody followed by a fluorescent secondary antibody over independent cycles. Detection therefore reflects protein abundance at one antigen site per assay. **b** COMET seqIF images showing spatial distribution of PD-1 (green) and PD-L1 (red) across a tumour-rich region of human melanoma tissue. **c** Violin plots of the distribution of PD-1 and PD-L1 pixel intensities within the tumour region in (**b**). **d** Schematic depiction of a two-site FuncOmap assay, where PD-1 and PD-L1 are labelled in the same cycle using donor- and acceptor-conjugated F(ab’2) fragments (green and red chromophores, respectively). When chromophores are within 1–10 nm, Förster resonance energy transfer (FRET) occurs. **e** Representative FuncOmap heatmap from a single acquisition region (512 × 512-pixel frame) captured by a semi-automated multiple-frequency-domain fluorescence lifetime imaging microscopy (mfFLIM). Pixel colour represents the FRET efficiency (Ef), reflecting the extent of PD-1/PD-L1 interaction. **f** Violin plot showing the heterogeneous distribution of Ef values sampled from the region shown in (**e**) (*n* = 1000 datapoints). The dotted line at 4% corresponds to the Förster radius (5.83 nm) below which interaction is not significant. **g** Conceptual illustration of interaction inference using expression-based profiling. PD-1^+^/PD-L1^+^ cells are annotated based on fluorescence intensity thresholds (90th percentile, P90) and potential interactions are inferred from co-localisation of PD-1^+^/PD-L1^+^ cells within a defined radius (70–100 nm). **h** Conceptual illustration of FuncOmap quantification of PD-1/PD-L1 interactions. PD-1/PD-L1 interactions are automatically quantified based on FRET between donor (PD-1) and acceptor (PD-L1) chromophores within 1–10 nm of each other.

The key differences in how traditional immunofluorescence (IF)-based assays and time-resolved-FRET-based FuncOmap analysis quantify immune checkpoint interaction have previously been described in detail [10, 11, 16] and are summarised in Fig. 1. Briefly, IF is an example of a one-site assay where PD-1 and PD-L1 are independently labelled using conventional primary and secondary antibodies over consecutive cycles (Fig. 1a). Conversely, FuncOmap is a spatial profile of a two-site assay where PD-1 and PD-L1 are labelled in the same cycle using conventional primary antibodies and species-specific F(ab’)2 fragments conjugated to either donor or acceptor chromophores (Fig. 1d) [10, 16].

An example of COMET seqIF DSP of PD-1 and PD-L1 expression in stage III metastatic melanoma tissue is shown in Fig. 1b. Regions of higher and lower PD-1 and PD-L1 expression are visually apparent, and cells are often classified as PD-1^+^ or PD-L1^+^ using fluorescence intensity thresholds, with interactions inferred from their spatial proximity [18, 19]. However, this binary classification does not capture the continuous variation in expression levels that influence receptor-ligand engagement. Consistent with this, the violin plot in Fig. 1c demonstrates a heterogeneous distribution of PD-1 and PD-L1 pixel intensities within an annotated tissue region.

FuncOmap enables spatial, per-pixel quantification of PD-1/PD-L1 interactions across tissue samples. These interactions were measured as FRET efficiency (Ef), where higher Ef values indicate close proximity and interaction between PD-1 and PD-L1 (Fig. 1g) [10]. A representative heatmap illustrates the spatial heterogeneity of Ef values within a single FuncOmap region (Fig. 1e), which is further quantified by its corresponding violin plot (Fig. 1f). Notably, Ef values below the Förster radius (*R*_0_) threshold (Ef = 4%; 5.83 nm) indicate a lack of interaction [10, 16, 20].

### COMET seqIF and FuncOmap (FuncO:TiME) analyses are coordinated using clinically annotated H&E staining

**Fig. 2:**
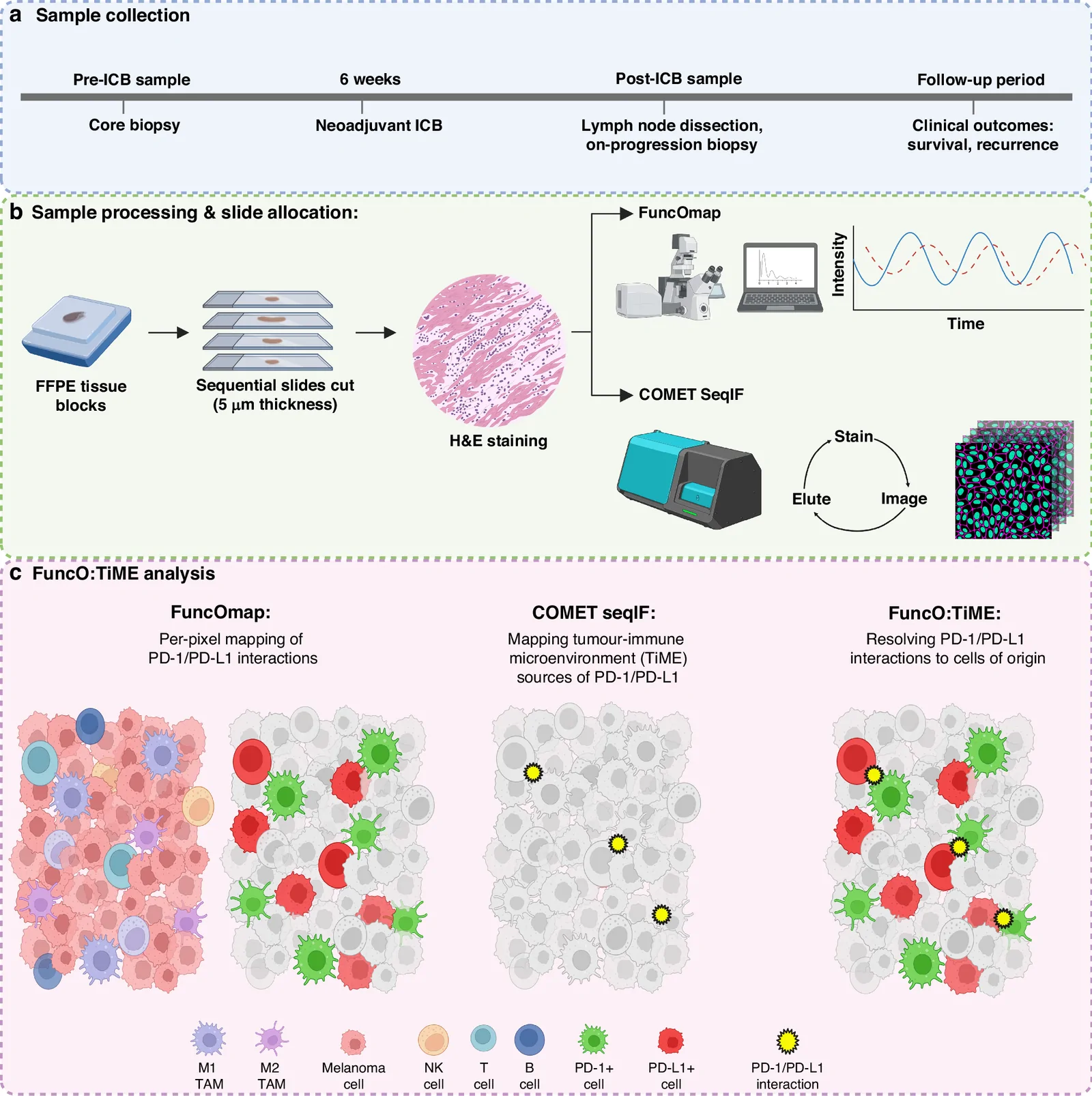
FuncO:TiME workflow integrating clinical sampling, spatial interaction mapping and immune cell profiling. **a** Clinical sampling timeline. Tumour samples are obtained from melanoma patients prior to neoadjuvant immune checkpoint blockade (ICB) and again following standard therapy (~2 cycles) at the time of lymph node dissection or on-progression biopsy. Clinical outcomes including recurrence and survival are assessed during follow-up. **b** Sample processing and slide allocation. Formalin-fixed paraffin-embedded (FFPE) tissue blocks are sectioned into sequential slides (5 µm thickness). Hematoxylin & Eosin (H&E)-staining is used for histologic annotation of tumour and non-tumour regions. Parallel sections are processed for FuncOmap acquisition and COMET seqIF to profile spatial immune cell populations through iterative staining, imaging, and antibody elution cycles. **c** FuncO:TiME computational integration. FuncOmap generates per-pixel maps of PD-1/PD-L1 interactions across tissue sections, while COMET seqIF identifies tumour and immune cells within the tumour-immune microenvironment (TiME). The FuncO:TiME framework integrates these datasets to resolve functional PD-1/PD-L1 interactions to their cellular sources within the TiME.

The overall workflow from patient sample collection to FuncO:TiME analysis is summarised in Fig. 2. Lymph node tissue samples from stage III metastatic melanoma patients were collected at baseline and after two cycles of neoadjuvant ICB treatment. H&E-stained sections were clinically annotated to define tumour and non-tumour regions, enabling coordinated spatial analysis across FuncOmap, COMET seqIF and downstream FuncO:TiME profiling.

 Mapping of FuncOmap regions to histologic features is illustrated in Supplementary Fig. 1. Time-resolved FRET images are acquired by a semi-automated multiple-frequency-domain FLIM. The different regions are mapped semi-automatically. Due to the semi-automatic motorised stage set-up, there is no bias from the operator during mapping and acquisition. The whole tissue is mapped. Images are processed using FuncOmap to generate pixel-wise FRET efficiency (Ef) maps, where each pixel represents the calculated PD-1/PD-L1 interaction state. Each of the Ef images is 512 × 512-pixels. These are mapped on the expression level of PD-L1. Therefore, FuncOmap contains both the expression state of PD-L1 and the interaction states of PD-1/PD-L1. FuncOmap-derived Ef data were computationally aligned to annotated H&E sections, allowing spatial localisation of PD-1/PD-L1 interactions within tissue architecture. This approach enabled region-specific integration of functional interaction data with histologic context. 

### FuncOmap highlights differences in the distribution of PD-1/PD-L1 interactions between tumour and non-tumour regions and pre- versus post-ICI treatment

This study represents the first application of the current, functionally advanced version of FuncOmap to whole lymph node tumour samples from stage III metastatic melanoma patients pre- and post-ICB treatment.

**Fig. 3:**
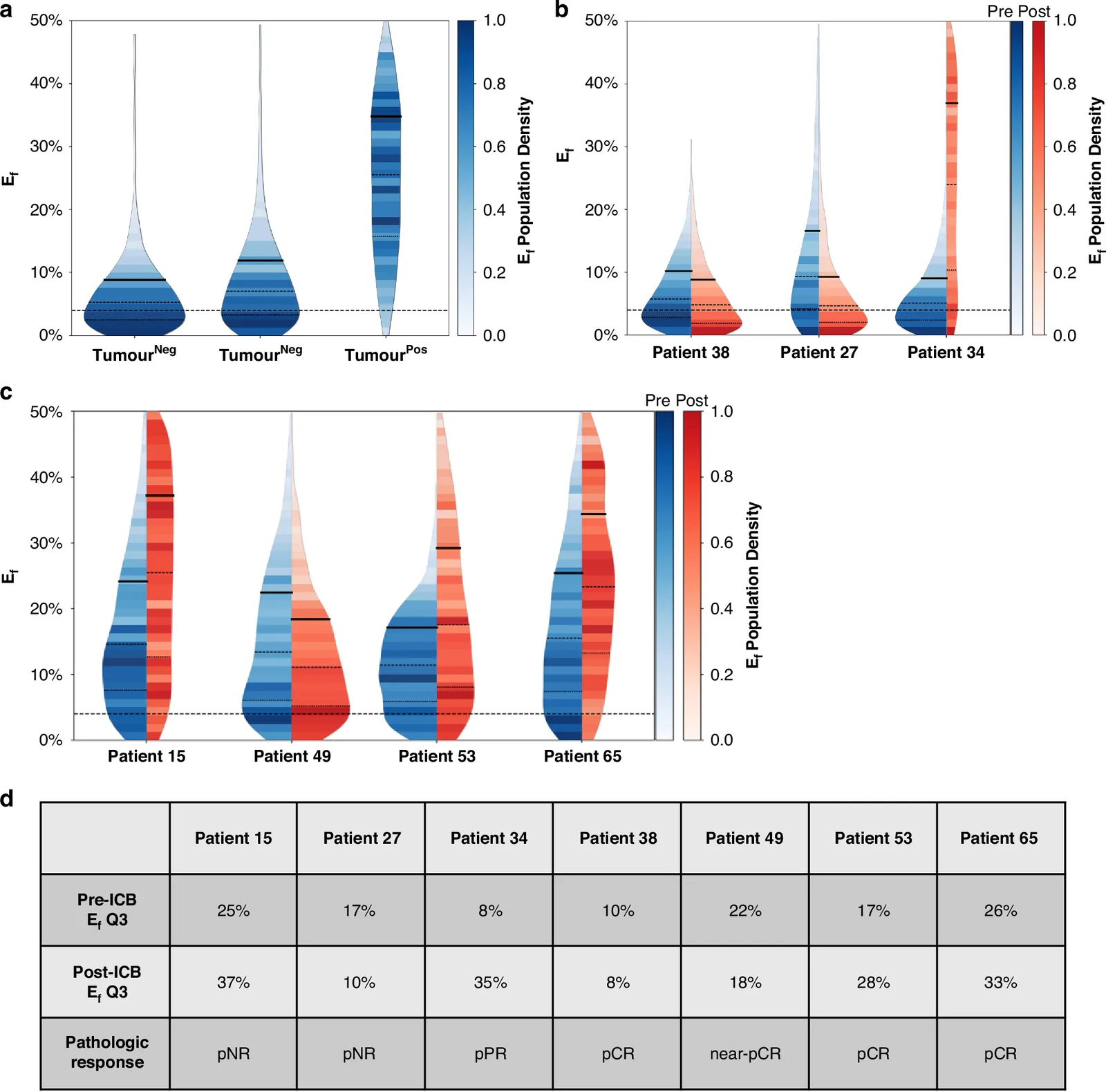
Global PD-1/PD-L1 interaction distributions across lymph node specimens before and after neoadjuvant immune checkpoint blockade (ICB). **a** Baseline PD-1/PD-L1 interaction efficiency (Ef) distributions across control lymph nodes. The first tumour-negative (tumour^neg^) sample represents a lymph node from a stage IB melanoma patient without nodal metastasis. The second tumour^neg^ and tumour-positive (tumour^pos^) samples derive from a separate patient with one negative and one metastatic sentinel lymph node, enabling within-patient comparison of interaction states between tumour-free and tumour-involved tissues. Violin plots represent the distribution of pixel-level Ef measurements across tissue sections. **b** Representative examples of global Ef distributions (30 × 10^6^ PD-1/PD-L1 Ef values) pre- (blue) and post- neoadjuvant ICB (red) in three melanoma patients with divergent pathologic responses (listed in **d**). **c** Additional patient samples showing pre- and post-ICB Ef distributions across metastatic melanoma lymph node specimens. Each violin plot represents the distribution of pixel-level Ef across the analysed tissue section. **d** Summary of Ef distribution changes across patients. The third quartile of the Ef distribution (Ef Q3) is shown for each patient pre- and post-ICB. Pathologic response classification is reported according to International Neoadjuvant Melanoma Consortium (INMC) criteria. (NR non-response, PR partial response, CR complete response, near-CR near-complete response). Within each violin plot, bold black lines indicate the Ef Q3, used as a representative summary of global PD-1/PD-L1 interaction states within each tissue sample. Dotted horizontal lines at 4% Ef correspond to the Förster Radius (*R*_0_), representing the approximate threshold for detectable FRET, below which is considered non-interaction.

In baseline lymph node samples from untreated stage IB melanoma patients undergoing routine sentinel lymph node biopsy (*n* = 2), PD-1/PD-L1 interaction levels varied across tissue regions, with higher Ef values observed in tumour-positive (tumour^pos^) compared to tumour-negative (tumour^neg^) (Q3 Ef 35% versus 9–11%; Fig. 3a).

Analysis of pre- and post-ICB lymph node tissues from stage III melanoma patients (*n* = 7) similarly revealed marked heterogeneity in PD-1/PD-L1 interactions across samples (Fig. 3b, c). However, no consistent differences in Q3 Ef values were observed across pathological response groups (CR, near-CR, PR, NR; Fig. 3d).

### FuncOmap confirms PD-1/PD-L1 interactive states cannot be inferred from PD-1/PD-L1 relative abundance alone

**Fig. 4:**
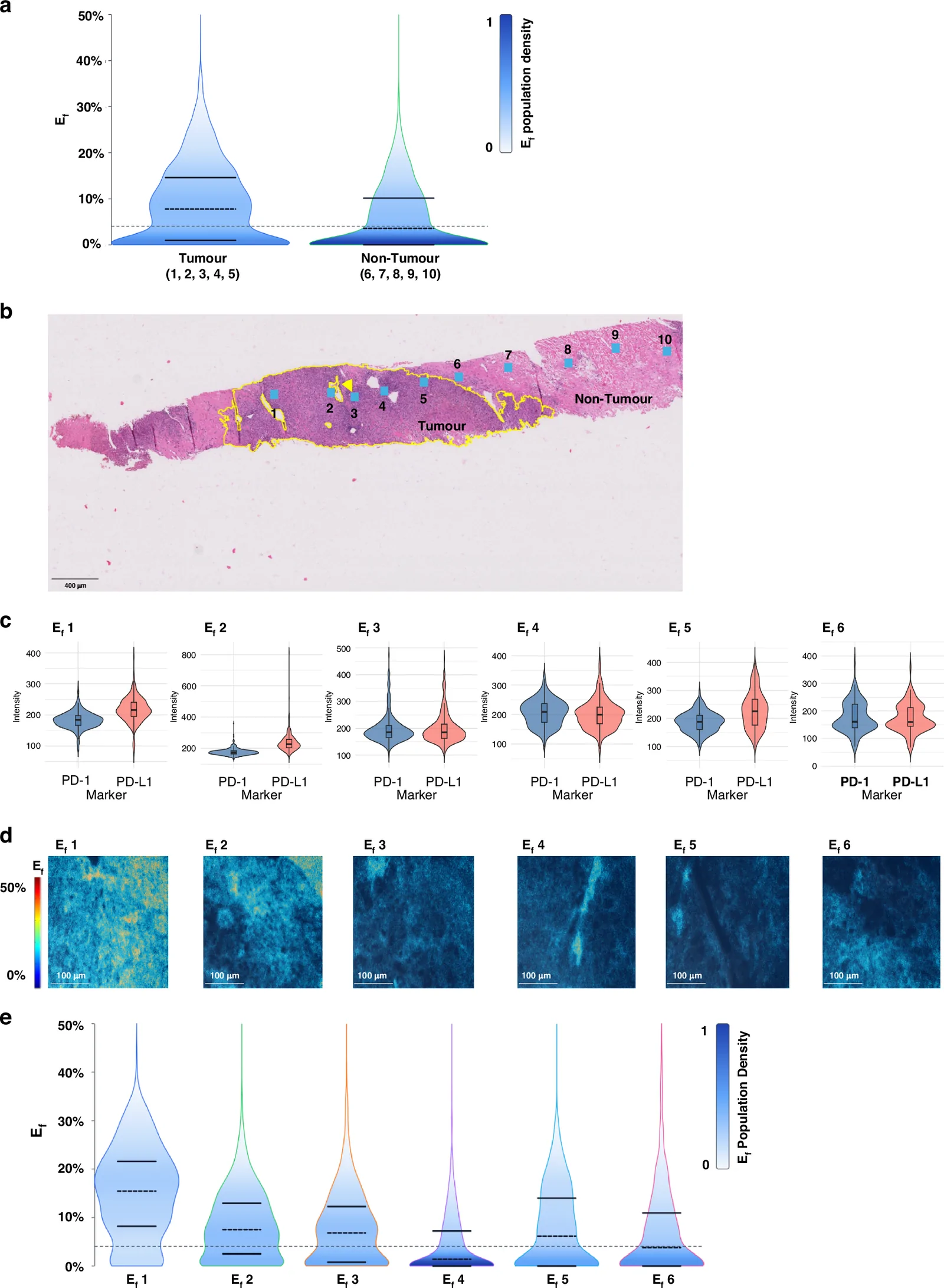
Spatial mapping of PD-1/PD-L1 interaction states across clinically annotated tumour and non-tumour regions using FuncOmap. **a** Violin plots showing the distribution of PD-1/PD-L1 interaction states, measured as FRET efficiency (Ef), across tumour-associated (regions 1–5) and non-tumour-associated (regions 6–10) tissue areas. Tumour-associated regions tended to exhibit higher Ef values and a greater density of elevated interaction states compared with non-tumour regions. **b** H&E-stained tissue section from a pre-treatment lymph node metastasis collected from a complete responder to immune checkpoint blockade (ICB) therapy. Tumour-positive (Tumour) and tumour-negative (Non-Tumour) regions were clinically annotated by histopathological assessment. Blue boxes represent the relative positioning of FuncOmap regions 1–10 (acquired at 63× magnification) on H&E tissue images (acquired at 20× magnification). Actual alignment of FuncOmap regions onto H&E images via FuncO:TiME computational workflow is described in the “Methods” section and Supplementary Fig. 1 (S1). **c** Violin plots showing PD-1 and PD-L1 expression distributions within individual FuncOmap regions (Ef 1–6). Regional variation in PD-1/PD-L1 interaction states was not consistently accompanied by corresponding differences in PD-1 or PD-L1 expression, illustrating that interaction states and checkpoint expression may provide distinct spatial information. **d** Representative FuncOmap heatmaps from regions Ef 1–6 quantifying the spatial distribution and density of PD-1/PD-L1 interaction states (Ef values) mapped onto PD-L1 expression. Ef values are coloured using a pseudo-colour scale where warmer colours indicate higher Ef values, whereas cooler colours indicate lower Ef values. **e** Region-specific violin plots showing the distribution of PD-1/PD-L1 interaction states (Ef) within individual FuncOmap regions. Distinct Ef distributions were observed across neighbouring tissue regions within the same specimen, highlighting the capacity of FuncOmap to visualise spatial variation in functional checkpoint interaction states at subregional resolution. Together, these analyses illustrate how FuncOmap can spatially localise PD-1/PD-L1 interaction states within clinically annotated tissue sections and provide a framework for investigating regional heterogeneity in functional checkpoint interactions beyond that captured by PD-1 or PD-L1 expression alone.

Following the observation of higher PD-1/PD-L1 interactions in tumour regions, pre-ICB lymph node tissue from a complete responder (patient 38) was analysed to assess spatial differences between tumour and non-tumour regions of a stage IIII melanoma sample (Fig. 4a, b). Consistent with stage IB samples, PD-1/PD-L1 interactions were higher in tumour compared to non-tumour regions (Q3 Ef 15% versus 11%; Fig. 4c).

To examine how these interaction patterns related to protein abundance, COMET seqIF analysis was performed on the same tissue, and PD-1/PD-L1 fluorescence intensity was quantified across FuncOmap regions (Fig. 4c). Comparison of PD-1/PD-L1 interactive states from FuncOmap imaging and PD-1/PD-L1 expression from COMET seqIF imaging confirmed that PD-1/PD-L1 interaction levels did not correspond to relative protein abundance (Fig. 4c–e). These findings suggest that PD-1/PD-L1 co-expression does not reliably reflect functional interaction, as captured by FRET-based FuncOmap analysis.

### FuncO:TiME profiling of PD-1/PD-L1 interactive regions captures region-specific differences in immune distribution and PD-1/PD-L1 expression

**Fig. 5:**
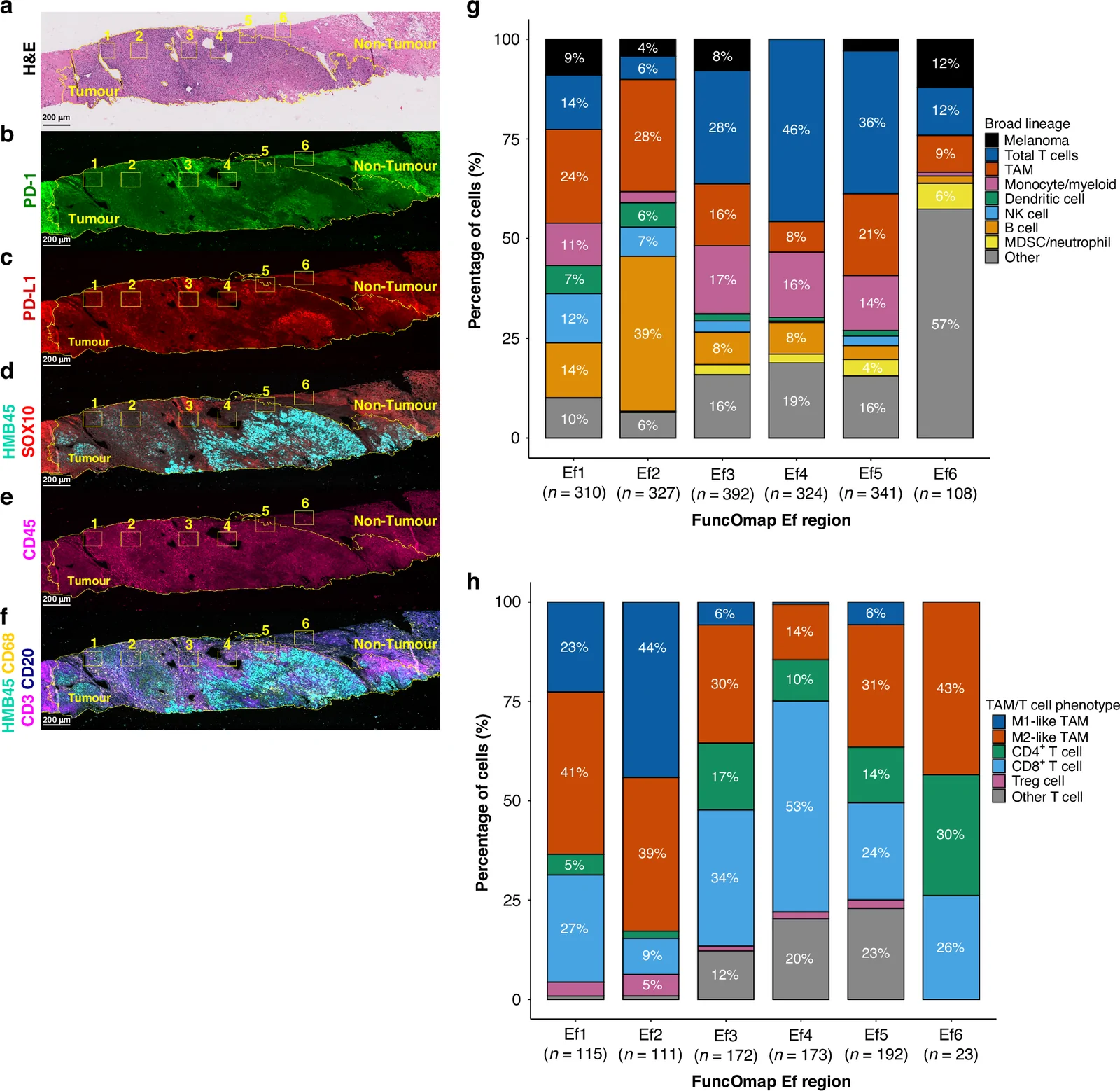
Spatial immune profiling of FuncOmap regions using the FuncO:TiME platform. **a** Representative H&E-stained section from a pre-treatment lymph node metastasis collected from a complete responder to immune checkpoint blockade (ICB) therapy. Tumour-positive (Tumour) and tumour-negative (Non-Tumour) tissue regions are outlined in yellow. Six manually annotated 512 × 512-pixel FuncOmap regions (1–6), as an example, were selected for high-resolution spatial profiling. Corresponding whole-slide FuncOmap images showing PD-1 (**b**) and PD-L1 (**c**) expression. Yellow boxes indicate the locations of FuncOmap regions 1–6 analysed in subsequent spatial profiling analyses. Representative COMET sequential immunofluorescence (seqIF) images of the same tissue section demonstrating expression of melanoma markers HMB45 and SOX10 (**d**), the pan-leucocyte marker CD45 (**e**), and a composite image showing HMB45, CD68, CD3, and CD20 (**f**). FuncOmap regions 1–6 are overlaid to illustrate their spatial relationship to tumour and immune cell distributions. **g** Broad immune lineage composition across FuncOmap regions. Stacked bar plots show the relative abundance of major cell populations identified by COMET seqIF, cell segmentation, and marker-based z-score lineage annotation, including total T cells, tumour-associated macrophages (TAMs), B cells, NK cells, dendritic cells, monocyte/myeloid populations, MDSC/neutrophils, melanoma cells, and unclassified (“Other”) cells. Percentages are calculated relative to all segmented cells within each FuncOmap region. **h ** TAM and T-cell phenotype composition across FuncOmap regions. Stacked bar plots show the relative abundance of TAM and T-cell phenotypes identified using marker-based z-score annotation, including M1-like TAMs, M2-like TAMs, CD4 T cells, CD8 T cells, regulatory T cells (Tregs), and other T-cell populations. Percentages are calculated relative to all phenotype-classified TAM and T-cell populations within each region.

Having established Ef region-specific differences in PD-1/PD-L1 interactive states, we next applied FuncO:TiME to examine how TiME cell distributions and their contributions to PD-1/PD-L1 expression varied across Ef regions. Higher PD-1/PD-L1 interaction patterns in FuncOmap region 1 (Ef 1) were associated with a fairly even distribution of immune lineages apart from tumour-associated macrophages (TAMs), whose proportions were slightly elevated in comparison with other lineages (Fig. 5g). FuncOmap regions Ef 2 (moderate PD-1/PD-L1 interaction levels) and Ef 4 (low interaction levels) were dominated by higher proportions of B cells and total T cells, respectively. FuncO:TiME analyses also permitted further subclassification of total TAM and T-cell populations, which also identified region-specific differences in pro-inflammatory (M1-like) and immunosuppressive (M2-like) TAMs and CD4+, CD8+ and regulatory T-cell populations (Fig. 5h).

**Fig. 6:**
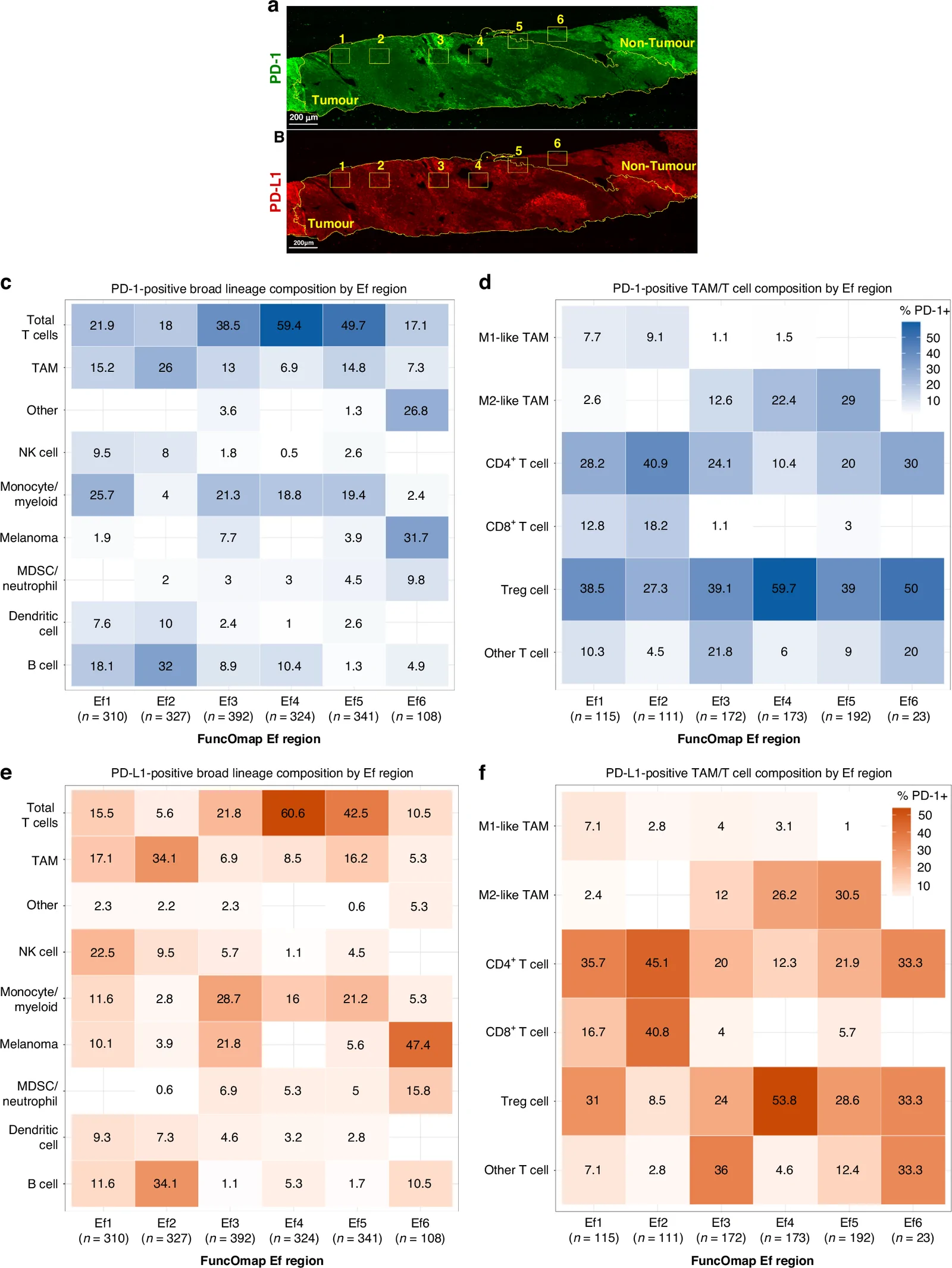
Spatial heterogeneity of PD-1- and PD-L1-expressing immune cell populations across FuncOmap regions. Representative whole-slide FuncOmap images showing PD-1 (**a**) and PD-L1 (**b**) expression within a pre-treatment lymph node metastasis from a complete responder to immune checkpoint blockade (ICB) therapy. Six manually annotated 512 × 512-pixel FuncOmap regions (1–6) are indicated and correspond to the regions analysed in Figs. 4 and 5. **c**, **d** Heatmaps showing the frequency of PD-1 expression across immune cell populations within each FuncOmap region. Broad immune lineage populations (**c**) and refined immune phenotypes (**d**) were identified using COMET sequential immunofluorescence (seqIF), cell segmentation, and marker-based z-score annotation. Values represent the percentage of cells within each lineage or phenotype that were classified as PD-1-positive in the corresponding FuncOmap region. **e**, **f** Heatmaps showing the frequency of PD-L1 expression across immune cell populations within each FuncOmap region. Broad immune lineage populations (**e**) and refined immune phenotypes (**f**) were identified using the same annotation workflow described above. Values represent the percentage of cells within each lineage or phenotype that were classified as PD-L1-positive in the corresponding FuncOmap region. PD-1 and PD-L1 positivity were defined using marker-specific z-score thresholds, and colour intensity reflects the relative frequency of PD-1-positive or PD-L1-positive cells within each lineage or phenotype. Together, these analyses demonstrate substantial regional heterogeneity in the cellular sources of PD-1 and PD-L1 expression across neighbouring FuncOmap regions and illustrate the ability of the FuncO:TiME workflow to resolve spatially localised immune checkpoint expression patterns within individual tumour specimens.

All immune lineages detected across each FuncOmap region were subsequently annotated for PD-1 and PD-L1 expression. COMET seqIF image panels and heatmaps highlighting the composition of PD-1- and PD-L1-expressing immune cell types are shown in Fig. 6. Similar to the heterogeneous distribution of immune lineages across FuncOmap regions, distinct patterns of PD-1- and PD-L1-expressing immune populations were observed (Fig. 6c, e). Ef 2 was characterised by a predominance of PD-1- and PD-L1-expressing B cells and TAMs, whereas PD-1/PD-L1 expression was primarily associated with total T cells and melanoma cells in Ef 4 and 6, respectively. In contrast, Ef 1 demonstrated a more even distribution of PD-1 and PD-L1-expressing cells across total T cells, TAMs, myeloid, NK and B cells. At the phenotypic level, M2-like TAMs and CD8^+^ T cells represented the predominant sources of PD-1/PD-L1 across FuncOmap regions, though Ef 2 and 3 showed more balanced distribution of M1-like versus M2-like TAM and CD4^+^ versus CD8^+^ T-cell contributions to PD-1/PD-L1 expression, respectively (Fig. 6d, f). Collectively, these findings highlight FuncO:TiME’s ability to contextualise spatial heterogeneity in PD-1/PD-L1 interactive states with immune cell-specific differences in PD-1/PD-L1 expression.

## Discussion

The introduction of immune checkpoint blockade (ICB) to melanoma treatment has transformed outcomes for patients with advanced melanoma, yet durable responses remain limited and predictive biomarkers for patient selection remain imperfect [21]. Immunohistochemistry (IHC)-based clinical scoring of PD-L1 expression classifies tumours according to the proportion of PD-L1^+^ cells. However, such approaches infer checkpoint interaction indirectly from protein abundance and do not account for the spatial distribution, relative abundance or functional interaction state of PD-1 and PD-L1 within the TiME; all of which influence receptor-ligand engagement [22, 23]. ICB acts by disrupting receptor-ligand interactions. Methods capable of directly resolving functional checkpoint interactions may provide biologically distinct information beyond expression-based assays.

In this study, we developed and applied a computational framework that integrates FuncOmap immune checkpoint interaction mapping with histologic annotation and digital spatial immune profiling to contextualise PD-1/PD-L1 interaction states within the TiME. Prior studies using intercellular FRET (iFRET) demonstrated that functional PD-1/PD-L1 interactions were detectable in tumours classified as PD-L1-negative by clinical assays and exhibited greater specificity than proximity-based methods such as PLA. However, these earlier iFRET approaches quantified interaction efficiency (Ef) without spatial localisation within tissue architecture. Functional Oncology mapping (FuncOmap) was therefore developed to extend iFRET analysis to spatially resolved tissue mapping, enabling regional characterisation of functional checkpoint interactions across the TiME at the nanometre scale [10].

Application of this approach to melanoma lymph node specimens demonstrated that PD-1/PD-L1 interactive states can diverge substantially from protein expression patterns. Regions with relatively low PD-1 or PD-L1 abundance sometimes exhibited higher interaction states than regions with higher protein expression, underscoring that checkpoint interaction cannot be reliably inferred from expression alone. These findings support the concept that functional receptor-ligand interactions represent a distinct biological parameter that may complement existing spatial proteomic approaches.

Using this framework, we compared global PD-1/PD-L1 interaction states across pre- and post-ICB lymph node tissues collected from stage III melanoma patients treated with neoadjuvant ICB and classified according to the International Neoadjuvant Melanoma Consortium (INMC) response criteria. Across patients, global interaction distributions did not segregate consistently by pathological response criteria (CR, near-CR, PR, NR). In several cases, PD-1/PD-L1 interaction levels increased following therapy, including 2 of 3 patients with CR despite absence of residual viable tumour. Given that pathological response categories are defined by differing levels of residual viable tumour post-ICB treatment, these results reinforce the need for FuncOmap to differentiate PD-1/PD-L1 interactive states across tumour versus non-tumour tissue areas. This finding motivates the need to spatially resolve what remaining TiME features and cellular components contribute to these interaction patterns.

To first differentiate PD-1/PD-L1 interactive states between tumour and non-tumour tissue regions, FuncOmap was integrated with clinically annotated Hematoxylin & Eosin (H&E) sections. These annotations were used to spatially assign FuncOmap acquisition regions to tumour and non-tumour tissue areas, enabling direct comparison of interactive states across distinct compartments of the TiME. Application of this approach to baseline lymph node tissues demonstrated higher PD-1/PD-L1 interaction levels within tumour-positive regions compared to tumour-negative regions. Similar compartment-specific differences were also observed in metastatic melanoma specimens. This improved FuncOmap methodology was applied, as an example, to pre-ICB tissue collected from patient 38 (CR). Once again, global PD-1/PD-L1 interactive states were higher in tumour regions compared to non-tumour regions.

Recent multimodal spatial analysis pipelines combining multiplex immunofluorescence, imaging mass cytometry, and histologic annotation have demonstrated the potential to integrate molecular and morphological features within tissue sections [24]. However, integrating imaging data across these modalities with differing optical properties and acquisition systems remains technically challenging. In contrast, the FuncOmap computational framework enables non-biased, semi-automated mapping of tens of millions (30 × 10^6^ per-pixel) nanoscale PD-1/PD-L1 interaction states across entire tissue sections and computational alignment with H&E images, enabling functional checkpoint interaction mapping within defined TiME compartments.

Although the H&E staining and FuncOmap analyses presented in this paper were performed on two sequential slides, both methods are non-destructive and enable spatial alignment of interaction measurements with histologic architecture. Future methodological refinements may help minimise the number of sequential slides required for multimodal integration with multiplex immune profiling platforms such as COMET seqIF. In this study, integration with multiplex immune profiling further demonstrated that PD-1/PD-L1 interaction states cannot be reliably inferred from relative protein abundance alone and provided orthogonal spatial immune profiling that independently contextualised FuncOmap interaction measurements across tumour compartments.

The distributions of PD-1/PD-L1 fluorescence intensities were quantified across the whole tumour region to approximate relative protein abundance. Comparison of PD-1/PD-L1 abundance and interactive state distributions revealed that higher PD-1/PD-L1 interaction states were observed in specific FuncOmap regions with seemingly lower PD-1/PD-L1 abundance. These observations reinforce the distinction between protein expression and functional receptor-ligand interaction and highlight the importance of directly measuring checkpoint interaction states when interpreting spatial immune signalling within the TiME. Direct measurement of checkpoint interaction may therefore provide complementary biological information to existing expression-based assays when interpreting immune checkpoint activity within tumour tissues. Integration with spatial immune profiling via FuncO:TiME revealed associations between regional PD-1/PD-L1 interaction states, immune cell composition and checkpoint protein expression in pre-ICB melanoma lymph node tissue. Distinct FuncOmap regions exhibited differing cellular sources of PD-1 and PD-L1, including T-cell-, TAM-, B cell- and melanoma-enriched patterns. However, these differences were not sufficient to fully explain the observed spatial heterogeneity in interaction states. As such, the current findings should be viewed as hypothesis-generating and support further development of the FuncO:TiME framework to directly resolve per-pixel PD-1/PD-L1 interaction states to single immune cell sources of PD-1/PD-L1 within the TiME. Where FuncO:TiME application to larger patient cohorts may validate specific immune populations as key sources of checkpoint interaction dynamics within the melanoma TiME.

Several limitations should be acknowledged. While global PD-1/PD-L1 interaction analyses were performed across multiple melanoma patient specimens, the integrated spatial immune profiling component of the FuncO:TiME workflow was conducted in a single pre-ICB lymph node specimen from a complete responder and should therefore be considered exploratory and hypothesis-generating. Consequently, the observed associations between regional PD-1/PD-L1 interaction states, immune cell composition, and checkpoint protein expression require validation in larger and more diverse patient cohorts. In addition, integration of FuncOmap and COMET seqIF data currently relies on manual transfer of annotations between sequential tissue sections using histopathological landmarks, which may introduce minor spatial alignment variability. Furthermore, because PD-1/PD-L1 interaction states and immune phenotyping were assessed on sequential rather than identical tissue sections, direct assignment of per-pixel FRET efficiency (Ef) values to individual PD-1- or PD-L1-expressing cells was not possible. Ongoing methodological development is focused on improving multimodal image registration and integrating FuncOmap, H&E, and multiplex immunofluorescence analyses across reduced numbers of sequential sections, with the goal of directly resolving checkpoint interaction states to specific cellular populations and enabling more comprehensive characterisation of the melanoma tumour-immune microenvironment.

## Conclusion

The FuncO:TiME framework provides a platform for spatially resolved mapping of functional checkpoint interactions within intact tumour tissues. By combining direct quantification of PD-1/PD-L1 molecular interactions with spatial immune profiling, FuncO:TiME enables investigation of how functional checkpoint interaction states relate to the cellular architecture of the TiME. While the present study establishes the feasibility of this integrated approach, future development will focus on directly resolving per-pixel interaction states to individual cell populations, improving multimodal image registration, and expanding analyses to larger clinically annotated patient cohorts. Together, these advances may facilitate a deeper understanding of checkpoint biology and support the development of functionally informed biomarkers for patient stratification and therapeutic response assessment.

## Supplementary information

Supplementary Tables and Figure.

Supplementary Methods_13_07_26.

## Data Availability

The data in this manuscript is not generated from existing sources. The FuncOmap data cannot be shared openly as FuncOmap, as explained in Methods section is under University of Bath Licence to Sam Legg, Dr. Julain Padget and Prof. Banafshé Larijani. Upon request to Julian Padget and Banafshé Larijani FuncOmap can be shared.
