# Supplementary material for: Development of functional spatial mapping of the tumour immune microenvironment (FuncO:TiME) in advanced melanomas
Source: BJC Rep. 2026 Sep 30;4:48. doi: 10.1038/s44276-026-00250-w (PMC13627726; doi:10.1038/s44276-026-00250-w)
Supplement: Supplementary file 1 [file 44276_2026_250_MOESM1_ESM.docx]

**Supplementary Table 1. Two-site Assays Used to Quantify Immune Checkpoint Interactions**

| **Reference:** | **One or Two-site assay(s) used:** | **Assay Range:** | **Protein Interaction quantified:** | **Does assay directly quantify functional interaction:** | **How are interactions scored:** |
| --- | --- | --- | --- | --- | --- |
| Naei, V.Y., Tubelleza, R., Monkman, J. et al. J Transl Med 23, 177 (2025). https://doi.org/10.1186/s12967-025-06186-y | Proximity Ligation Assay (PLA) | Within 40nm | PD-1/PD-L1 | No, proximity of PD-1+ and PD-L1+ cells within 40nm are inferred as interacting | Interactions classified as positive or negative based on PD-1/PD-L1 intensity thresholds |
| Roche VENTANA PD-L1 (SP142) assay | One-site Immunohistochemistry (IHC) for PD-L1 | 70-100nm | N/A | No, the proportion of tumor (TC) or immune (IC) cells are scored as the proportion of viable cells showing PD-L1 staining of any intensity | N/A |
| Veeriah S., Leboucher P., de Naurois J. et al. Cancer Res (2014) 74 (18): 4983–4995. https://doi.org/10.1158/0008-5472.CAN-13-3382 | Coincidence amplified intracellular Förster Resonance Energy Transfer (aFRET) | 1-10nm | Intracellular Activation of protein kinase B (Akt) by Phosphorylation | No, quantifies intracellular Akt activation status based on interaction between pan-Akt and phospho-Akt antibodies within 10nm | N/A |
| Sánchez-Magraner L., Miles J., Baker C. L. et al.  Cancer Res (2020) 80 (19): 4244–4257.  https://doi.org/10.1158/0008-5472.CAN-20-1117 | Intercellular/immune Förster Resonance Energy Transfer (iFRET) and PLA | 1-10nm, 17-40nm | PD-1/PD-L1, CTLA-4/CD80 | PLA: no, see above  iFRET: yes, PD-1/PD-L1 interactions based on chromophore energy transfer between cells within 10nm | Median FRET efficiency (Ef) across tissues |
| Sánchez-Magraner L., Gumuzio J., Miles J., et al. J Clin Oncol (2023) 41 (14): 2561-2570. https://doi.org/10.1200/JCO.22.01748 | QF-Pro (based on iFRET) | 1-10nm | PD-1/PD-L1 | Yes, see above | Median Ef values across tissues |
| Sarygina E, Applebee C., McIntyre A., et al. BJC Reports (2024) 2(1):1-11. https://doi.org/10.1038/s44276-023-00033-7 | Functional Oncology Mapping (FuncOmap) | 1-10nm | PD-1/PD-L1 | Yes, iFRET coupled to a computational algorithm for automated Ef thresholding. | Per-pixel Ef values per coincidence regions across patient tissue samples |

| **Patient #** | **Gender** | **Age**  **(Years)** | **Ethnicity** | **Prior Therapy** | **Neoadjuvant**  **ICI Regimen**  **Received** | **Cycles Given** | **TNM Stage** | **AJCC Stage** | **Pathological**  **Response** | **RVT (%)** | **Tumor Necrosis**  **(%)** | **Tumor Melanosis/Fibrosis (%)** | **PFS** | **OS** | **Status** |
| --- | --- | --- | --- | --- | --- | --- | --- | --- | --- | --- | --- | --- | --- | --- | --- |
| 15 | F | 69 | Hispanic | No | Anti-PD-1 + Anti-LAG3 | 2 | pT3b, pN2b, cM0 | IIIC | NR | 70 | 25 | 25 | 63 | 63 | Alive |
| 27 | M | 59 | Non-Hispanic | No | Anti-PD-1 + Anti-LAG3 | 2 | pT1a, cN2b, cM0 | IIIB | NR | 60 | 35 | 5 | 62 | 62 | Alive |
| 34 | F | 74 | Non-Hispanic | No | Anti-PD-1+ Anti-CTLA4 | 5 | pT4b, pN2a, pMx | IIIC | PR | 50 | N/A | N/A | 26 | 36 | Deceased |
| 38 | M | 71 | Non-Hispanic | No | Anti-PD-1 + Anti-LAG3 | 2 | cN2b, cM0 | IIIB | Near-CR | 3 | 10 | 87 | 61 | 61 | Alive |
| 49 | M | 51 | Non-Hispanic | No | Anti-PD-1 + Anti-LAG3 | 1 | pT4b, pN1c, pMx | IIIC | Near-CR | 5 | <5 | 92 | 50 | 50 | Alive |
| 53 | F | 57 | Non-Hispanic | No | Anti-PD-1 + Anti-LAG3 | 2 | pT1b, cn2b, cM0 | IIIB | CR | 0 | 70 | 30 | 45 | 45 | Alive |
| 65 | M | 71 | Non-Hispanic | No | Anti-PD-1 + Anti-LAG3 | 3 | rpT0, pN1b, cM0 | IIIB | CR | 0 | <5 | 95 | 46 | 46 | Alive |

**Supplementary Table 2. Clinicopathologic Features of Patients:** TNM and AJCC stages were categorized according to the American Joint Committee on Cancer (AJCC) (8^th^ Edition). Pathologic Responses were categorized according to International Neoadjuvant Melanoma Consortium (INMC) guidelines. Abbreviations: RVT; Residual Viable Tumor, PFS; Progression Free Survival, OS; Overall Survival.

**Supplementary Figure 1. Overview of FuncOmap region alignment to H&E tissues via the FuncO:TiME computational workflow:** (1) FLIM-FRET lifetime images are processed using FuncOmap to generate pixel-wise FRET efficiency (Ef) maps, where each pixel represents the calculated PD-1/PD-L1 interaction state. (2) Individual FLIM sampling regions across entire tissues are computationally registered to the corresponding H&E-stained tissue section through global alignment followed by local refinement, enabling spatial correspondence between functional interaction maps and tissue histology. (3) Registered sampling regions can be interrogated individually to visualize regional Ef heatmaps together with local Ef value distributions, allowing comparison of functional checkpoint interaction states across distinct histologic regions. In this study, selected FuncOmap regions were used to compare regional variation in PD-1/PD-L1 interaction states between adjacent tumor-positive and tumor-negative tissue areas. Complete details of the computational registration and analysis workflow are provided in the Methods.
